# Oral corticosteroid dosage and taper duration at onset in myelin oligodendrocyte glycoprotein antibody-associated disease influences time to first relapse

**DOI:** 10.1136/jnnp-2024-333463

**Published:** 2024-05-14

**Authors:** Benjamin P Trewin, Russell C Dale, Jessica Qiu, Melissa Chu, Niroshan Jeyakumar, Fionna Dela Cruz, Jane Andersen, Pakeeran Siriratnam, Kit Kwan M Ma, Todd A Hardy, Anneke van der Walt, Jeanette Lechner-Scott, Helmut Butzkueven, Simon A Broadley, Michael H Barnett, Stephen W Reddel, Fabienne Brilot, Tomas Kalincik, Sudarshini Ramanathan, On behalf of the Australasian MOGAD Study Group, Robert Adam, Ian Andrews, Jayne Antony, Patrick Aouad, Monica Badve, Joshua Barton, Heidi Beadnall, Stefan Blum, Michael Boggild, David A Brown, Jim Burrow, Katherine Buzzard, Ann Bye, Anita Cairns, Sophie Calvert, Shabeed Chelakkadan, Damian R Clark, Marzena J Fabis-Pedrini, Deborah Field, Anthony Fok, Clare L Fraser, Victor SC Fung, Justin Garber, Serge Geara, Deepak Gill, Sachin Gupta, Simon Hawke, Andrew PD Henderson, Nevin A John, Dean L Jones, Hannah F Jones, Allan Kermode, Matthew Kiernan, Trevor Kilpatrick, Andrew J Kornberg, Mitchell Lawlor, Fiona XZ Lee, Richard J Leventer, Vivien Li, Simon Ling, Ganesha Liyanage, Joseph A Lopez, Stephen Malone, Mark P Marriot, Pamela McCombe, Alan McDougall, Manoj P Menezes, Vera Merheb, Christina Miteff, Mastura Monif, Gopinath Musuwadi Subramanian, Ai-Lan Nguyen, Gina O’Grady, John O’Neill, Robert Ouvrier, Mark Paine, John Parratt, Sekhar Pillai, Jane Prosser, Sean DS Riminton, Izanne Roos, Jennifer Sandbach, Ingrid E Scheffer, Ubaid Shah, Neil Shuey, Adriane Sinclair, Mark Slee, Claire G Spooner, Ian Sutton, Sanjay Swaminathan, Esther Tantsis, James Thomas, Terrence Thomas, Julia Thompson, Christopher Troedson, Steve Vucic, Justine Wang, Tyson Ware, Richard Webster, Ming Wei Lin, Owen White, Wei Yeh, Con Yiannikas, Eppie M Yiu, Michael Zong

**Affiliations:** 1 Translational Neuroimmunology Group, Kids Neuroscience Centre and Brain and Mind Centre, Sydney Medical School, Faculty of Medicine and Health, University of Sydney, Sydney, New South Wales, Australia; 2 Clinical Neuroimmunology Group, Institute for Neuroscience and Muscle Research, Kids Research Institute at the Children's Hospital at Westmead, University of Sydney, Sydney, New South Wales, Australia; 3 Sydney Medical School, Faculty of Medicine and Health, University of Sydney, Sydney, New South Wales, Australia; 4 Department of Medicine, The University of Melbourne, Melbourne, Victoria, Australia; 5 Department of Neurology, The Royal Melbourne Hospital, Parkville, Victoria, Australia; 6 Brain Autoimmunity, Kids Neuroscience Centre, Kids Research at the Children's Hospital at Westmead, Sydney, New South Wales, Australia; 7 Department of Neuroscience, Monash University Central Clinical School, Melbourne, Victoria, Australia; 8 Department of Neurology, Concord Hospital, Concord, New South Wales, Australia; 9 Alfred Hospital, Melbourne, Victoria, Australia; 10 The University of Newcastle, Newcastle, New South Wales, Australia; 11 School of Medicine, Griffith University, Nathan, Queensland, Australia; 12 Department of Neurology, Gold Coast University Hospital, Southport, Queensland, Australia; 13 Brain and Mind Centre, The University Of Sydney, Camperdown, New South Wales, Australia; 14 School of Medical Science, Faculty of Medicine and Health, University of Sydney, Sydney, New South Wales, Australia

**Keywords:** immunology, myelin, neuroimmunology, steroids

## Abstract

**Background:**

We sought to identify an optimal oral corticosteroid regimen at the onset of myelin oligodendrocyte glycoprotein antibody-associated disease (MOGAD), which would delay time to first relapse while minimising cumulative corticosteroid exposure.

**Methods:**

In a retrospective multicentre cohort study, Cox proportional hazards models examined the relationship between corticosteroid course as a time-varying covariate and time to first relapse. Simon-Makuch and Kaplan-Meier plots identified an optimal dosing strategy.

**Results:**

We evaluated 109 patients (62 female, 57%; 41 paediatric, 38%; median age at onset 26 years, (IQR 8–38); median follow-up 6.2 years (IQR 2.6–9.6)). 76/109 (70%) experienced a relapse (median time to first relapse 13.7 months; 95% CI 8.2 to 37.9). In a multivariable model, higher doses of oral prednisone delayed time to first relapse with an effect estimate of 3.7% (95% CI 0.8% to 6.6%; p*=*0.014) reduced hazard of relapse for every 1 mg/day dose increment. There was evidence of reduced hazard of relapse for patients dosed ≥12.5 mg/day (HR 0.21, 95% CI 0.07 to 0.6; p*=*0.0036), corresponding to a 79% reduction in relapse risk. There was evidence of reduced hazard of relapse for those dosed ≥12.5 mg/day for at least 3 months (HR 0.12, 95% CI 0.03 to 0.44; p*=*0.0012), corresponding to an 88% reduction in relapse risk compared with those never treated in this range. No patient with this recommended dosing at onset experienced a Common Terminology Criteria for Adverse Events grade >3 adverse effect.

**Conclusions:**

The optimal dose of 12.5 mg of prednisone daily in adults (0.16 mg/kg/day for children) for a minimum of 3 months at the onset of MOGAD delays time to first relapse.

**WHAT IS ALREADY KNOWN ON THIS TOPIC**

**WHAT THIS STUDY ADDS**

- In this multicentre cohort study of 109 children and adults with MOGAD, there was evidence that patients treated with at least 12.5 mg/day (0.16 mg/kg in children) of oral prednisone for at least 3 months had an 88% reduction in the risk of relapse compared with those who did not receive this regimen.

**HOW THIS STUDY MIGHT AFFECT RESEARCH, PRACTICE OR POLICY**

- This study highlights the need for effective early treatment after MOGAD onset to modify the long-term disease trajectory, and for the first time provides recommendations to clinicians on the corticosteroid dosing regimen and duration at disease onset to minimise relapsing disease, while minimising cumulative corticosteroid exposure. This study represents an important step forward in improving treatment and outcomes for children and adults with MOGAD.

## Introduction

 Myelin oligodendrocyte glycoprotein antibody-associated disease (MOGAD) is a distinct antibody-associated demyelinating disorder.^1 2^ MOGAD commonly presents as bilateral or recurrent optic neuritis (ON) and transverse myelitis (TM) in children and adults, and acute disseminated encephalomyelitis (ADEM) in children.^3^,^5^ Less common manifestations encompass brainstem or cerebellar involvement, cortical encephalitis and leptomeningeal involvement.^4^,^6^ Demyelination due to MOGAD has the potential to cause permanent visual, motor and sphincter dysfunction.^47^,^9^ The recent publication of consensus diagnostic criteria represents an important step in expediting early recognition of this condition.^10^

We previously identified MOGAD as a condition that exhibits significant and often rapid responsiveness to corticosteroids and can be corticosteroid dependent.^3 4^ Relapses were associated with rapid tapering of oral prednisone at doses <10 mg daily or within the first 2–3 months following cessation.^3 4^ Subsequent studies have supported the modulating effect of corticosteroids on inducing remission and identified longer courses of treatment may be associated with reduced relapses with suggested treatment durations ranging from 5 weeks to 6 months.^11^,^13^ There is emerging evidence that reduced relapse rates in the first year after onset may be associated with a reduction in the long-term relapse risk.^14^ Such insights magnify the critical nature of interventions during this nascent phase and suggest therapeutic decisions at onset might influence subsequent disease course. Yet, the well-documented impact of long-term corticosteroid exposure encompassing neuropsychiatric, metabolic and bone health-related adverse effects across the ages,^15^ underscores the complexity of therapeutic decision-making in MOGAD.

At present, there is equipoise regarding optimal therapeutic approaches in MOGAD, with variable clinician preference and experience.^16^ While retrospective studies and systematic reviews report intravenous immunoglobulin and rituximab may reduce activity in relapsing MOGAD,^17^,^19^ there is currently no consensus on optimal treatment at onset. Our objective was to explore potential therapeutic strategies for MOGAD patients at disease onset in a retrospective cohort, by evaluating whether a specific oral prednisone regimen could effectively delay time to first relapse (TTFR), while minimising cumulative corticosteroid exposure.

## Materials and methods

### Study design and participants

This was a retrospective, observational, multicentre cohort study of 24 centres from the Australasian MOGAD Study Group. Patients were included if they were seropositive for MOG immunoglobulin G by a live cell-based assay and had a clinical phenotype compatible with MOGAD as per proposed diagnostic criteria.^10^ Patients were required to have detailed clinical, therapeutic (including dose and timing of corticosteroids and other treatment), and outcome (including Expanded Disability Status Scale and visual acuities) data available, and a minimum follow-up of 12 months. An investigator-led national MOGAD REDCap electronic data registry was developed in 2020. Predetermined fields relevant to this study were identified, and required data were extracted from detailed review of clinical information.

An onset clinical episode or relapse was defined as new focal or multifocal central nervous system symptoms and/or signs lasting at least 24 hours, supported clinically and/or radiologically as attributable to MOGAD, in the absence of concurrent infection and following exclusion of an alternate diagnosis. A relapse included neurological symptoms and/or signs separated from initial presentation by a period of recovery of at least 2 weeks.

Patients ≤16 years of age at onset were classified as paediatric. Doses of intravenous and oral corticosteroids in children were evaluated in conjunction with adult patients using a weight-based conversion, where each paediatric patient’s dose was multiplied by the ratio of the median weight of an Australian adult (76.35 kg)^20^ to the patient’s weight at dosing.^21^ All corticosteroid doses were converted into the equivalent dose of prednisone.^22^

‘Acute therapy’ refers to treatment within the first month of a clinical episode including pulsed intravenous corticosteroids, intravenous immunoglobulin induction or plasmapheresis. ‘Maintenance therapy’ is defined as a non-corticosteroid treatment continued for ≥6 months.

### Study outcomes

**Figure 1:**
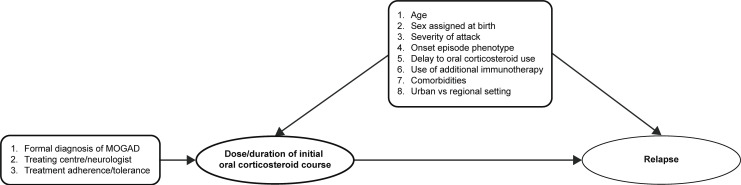
Directed acyclic graph examining potential variables that may influence the relationship between oral corticosteroid treatment and time to first relapse. Pseudorandomising factors (on the left) are variables that lead to heterogeneity in the exposure of interest (dose/duration of initial oral corticosteroid course). Potentially confounding covariates (above) are also listed with their relationship to exposure and outcome (time to first relapse). MOGAD, myelin oligodendrocyte glycoprotein antibody-associated disease.

The exposure of interest was the daily dosage of oral corticosteroids following disease onset until first relapse or last follow-up, whichever occurred first. The primary outcome was TTFR. Secondary outcomes included annualised relapse rate, disease course at last follow-up and adverse event frequency as defined by Common Terminology Criteria for Adverse Events grade (CTCAE V.5.0).^23^ The potential variables affecting the relationship between oral corticosteroids and TTFR are highlighted in a directed acyclic graph (figure 1).

### Statistical analyses

Descriptive summary statistics were used for demographics, clinical characteristics and treatment details. Medians and IQRs were calculated for continuous variables, and frequencies and percentages were calculated for categorical variables. The standardised mean difference (SMD) was used to examine the balance of covariate distribution between children and adults.

The relationship between oral corticosteroid dose and TTFR was visualised in Simon-Makuch survival plots, which allow for a time-dependent covariate (oral corticosteroid dose) to be used as a stratifying variable.^24^ The association between the dose of oral corticosteroids and TTFR was analysed using Cox proportional hazards models adjusted for potential confounding variables, identified based on plausible contribution (as outlined in figure 1) or a univariate association at α≤0.1.

The relationship between time spent above the optimal oral corticosteroid dose and risk of a relapse was visualised in a series of Kaplan-Meier curves using different duration cut-offs to categorise patients into groups. A subgroup analysis was planned to assess for the presence of a differential response between children and adults. The annualised relapse rate during each of the first 6 months from MOGAD onset was compared between the cumulative time that patients spent treated with oral corticosteroids above versus below the nominated threshold oral corticosteroid dose.

Patients with a follow-up <12 months were excluded. The only variable with missing data referred specifically to onset episode severity in 20/109 patients. When stratifying this variable, one stratum was designated for missing data, which enabled us to include it in the multivariable analysis.

Statistical analyses were performed with R statistical software (V.4.3.1).^25^

## Results

Between 1 July 2009 and 31 August 2023, 745 patients tested positive for MOG IgG using a flow cytometry live cell-based assay as previously described.^26 27^ Detailed clinical data meeting predefined inclusion criteria was available from 109 patients.

Demographics, disease duration and course, initial episode phenotype and immunotherapy in 109 patients with MOGAD are summarised in table 1. Patients had a median age of onset of 26 years (IQR 8–38). 41/109 (38%) were paediatric onset, 62/109 (57%) were assigned female at birth, 76/109 (70%) experienced a relapsing course and median duration of follow-up was 6.2 years (IQR 2.6–9.6). Sex assigned at birth, a relapsing disease course and duration of follow-up were similar between the paediatric and adult groups (SMD<0.14). Patients who had not experienced relapse at the last follow-up had a shorter median duration of follow-up (2.6 years, IQR 2–4.6), compared with those who had relapsed (6.9 years, IQR 4.8–10.3, SMD=0.75).

**Table 1:** Demographic, clinical, therapeutic and outcome data in the total cohort and stratified by age

		Total cohort	Paediatric onset	Adult onset	Standardised mean difference
n		109	41	68	–
Onset age, years median (IQR)		26 (8–38)	7 (5–9)	34 (29–46)	2.99
Sex assigned at birth, n females (%)		62 (57)	25 (61)	37 (54)	0.13
Disease course, n of relapsing patients (%)		76 (70)	28 (68)	48 (71)	0.05
Duration of follow-up, years median (IQR)		6.2 (2.6–9.6)	5.9 (2.7–10.1)	6.3 (2.6–9.2)	0.02
Onset episode phenotypen of patients (%)	Isolated optic neuritis (% of total phenotypes)	58 (53)	15 (37)	43 (63)	0.55
	Bilateral (n)	35	10	25	0.26
	Unilateral (n)	23	5	18	–
	Isolated transverse myelitis (TM) (% of total cohort)	10 (9)	1 (2)	9 (13)	0.38
	Longitudinally extensive (n)	4	1	3	0.1
	Short segment only (n)	6	0	6	–
	Acute disseminated encephalomyelitis	19 (17)	19 (46)	0	1.5
	Cerebral cortical encephalitis	2 (2)	1 (2)	1 (1)	0.07
	Mixed phenotypes*	19 (17)	5 (12)	14 (21)	0.22
Onset episode treatmentn of patients (%)	Nil	18 (17)	5 (12)	13 (19)	0.19
	IV corticosteroids only	26 (24)	4 (10)	22 (32)	0.54
	Oral corticosteroids only	12 (11)	6 (15)	6 (9)	0.18
	IV and oral corticosteroids	53 (49)	26 (63)	27 (40)	0.48
	Initial oral corticosteroid dose (mg/kg)	0.96 (0.65–1.31)	1.28 (1.04–1.92)	0.65 (0.65–0.65)	1.78
	Corticosteroids and IVIg/PLEX	9 (8)	5 (12)	4 (6)	0.23
Maintenance treatment instituted at disease onset	IVIg/PLEX	0	0	0	–
	Other immunotherapies†	5 (5)	0	5 (7)	0.35

Data are summarised as n (%), or median (IQR). disseminated encephalomyelitis.

* Other presentation phenotypes: 1 UON+STM (adult); 1 UON+LETM (paediatric); 1 BON+LETM (adult);1 UON+STM+area postrema syndrome (adult); 1 BON+LETM+diencephalic+Iintracranial hypertension; 4 Ccerebral syndrome (adults); 1 BON+LGI1 antibody encephalitis (adult); 1 ADEM+BON (paediatric); 1 ON+cerebellum (adult); 1 TM+cerebellum (adult); 1 LETM+Ccerebral (adult); 2 LETM+brainstem (adults); 1 CCE+brainstem (adult); 1 brainstem+cerebellum (paediatric); 1 Aaseptic meningitis (adult).

† Other maintenance immunotherapies: 3 rituximab; 11 azathioprine; 11 mycophenolate mofmofetil.

ADEMacute disseminated encephalomyelitisBON, bilateral optic neuritis; CCE, cerebral cortical encephalitis; IVintravenousIVIg, Intravenous immunoglobulin; LETM, longitudinally extensive transverse myelitis; LGI1, Leucine rich glioma-inactivated 1; PLEX, Therapeutic plasma exchange; STM, short transverse myelitis; UON, unilateral optic neuritis

The most common presentation phenotype was isolated ON in 58 patients (53%), followed by isolated ADEM in 19 (17%), and then isolated TM in 10 (9%) (table 1). 35 of 58 ON patients were clinically bilateral at onset. There was a higher incidence of isolated ON at onset for adults compared with children (63% vs 37%, SMD=0.26). ADEM was exclusively identified in paediatric patients.

18/109 (17%) patients did not receive any immunotherapy at onset while 91/109 (83%) received oral and/or intravenous corticosteroids (table 1). Nine (8%) patients received intravenous immunoglobulin and/or plasmapheresis as acute treatment, in addition to corticosteroids. Intravenous corticosteroids in isolation were more commonly used in adults (children 10%, adults 32%, SMD=0.54) while a combination of intravenous and oral corticosteroids was more frequently administered in children (children 63%, adults 40%, SMD=0.48). Maintenance immunotherapy outside of intravenous immunoglobulin and plasmapheresis instituted for the first clinical episode was used in a minority of adults (7%; rituximab n=3, azathioprine n=1, mycophenolate n=1) and in none of the children.

### Timing of clinical episodes relative to corticosteroids

76 relapses occurred in 109 patients with a median survival TTFR of 13.7 months (95% CI 8.2 to 37.9). The relationship between oral prednisone courses and the first relapse was evaluated. 40/65 patients treated with oral prednisone went on to experience a relapse (online supplemental figure 1), of which 21/40 relapsed on prednisone doses <10 mg/day or within 10 weeks of cessation. The median time from first symptoms of the onset episode to initiation of oral prednisone was 13 days (IQR 9–22 days) and the median oral prednisone course duration was 47 days (IQR 30–140). The rate of steroid taper is visualised in online supplemental figure 2.

### Multivariable modelling of TTFR

The univariate and multivariable associations between patient and disease characteristics, and cumulative hazard of a relapse are shown in table 2. The univariate models identified that higher oral prednisone doses were associated with a lower risk of a relapse (HR 0.96 (95% CI 0.93 to 0.99); p*=*0.0078). The univariate models did not provide evidence for an association with other covariates including onset age, sex assigned at birth, onset phenotype (inclusive of ON, TM and ADEM presentations), time from onset to initiation of corticosteroids, episode severity, the use of intravenous corticosteroids inclusive of total dosage or concurrent intravenous immunoglobulin or plasmapheresis. This association was confirmed in a multivariable model (HR 0.96 (95% CI 0.93 to 0.99); p*=*0.014) adjusted for onset age, sex, time from onset to initiation of prednisone and episode severity. The estimated effect size was a 3.7% (95% CI 0.8% to 6.6%) reduction in the hazard of a relapse for every 1 mg/day increment in oral prednisone dose. This equated to a 17.2% (95% CI 3.8% to 28.8%) reduction in the hazard of a relapse for every 5 mg/day oral prednisone dose increment.

**Table 2:** Univariate and multivariable Cox proportional hazard models of time to first relapse

Covariate		n	Univariate		Multivariable	
			HR (95% CI)	P value	HR (95% CI)	P value
Onset age (years)		109	1 (0.99 to 1.01)	0.80	1 (0.98 to 1.01)	0.48
Sex assigned at birth	Female	62	1.00 (reference)	–	1.00 (reference)	–
	Male	47	1.25 (0.79 to 1.97)	0.34	1.45 (0.9 to 2.34)	0.13
Initial phenotype	No ON	51	1.00 (reference)	–	–	–
	ON	58	1.24 (0.78 to 1.96)	0.36	–	–
	No TM	100	1.00 (reference)	–	–	–
	TM	10	0.54 (0.2 to 1.48)	0.23	–	–
	No ADEM	90	1.00 (reference)	–	–	–
	ADEM	19	0.83 (0.45 to 1.54)	0.56	–	–
Onset episode severity	EDSS<3	18	1.00 (reference)	–	1.00 (reference)	–
	EDSS≥3	71	0.71 (0.39 to 1.3)	0.27	0.75 (0.38 to 1.46)	0.40
	Missing	20	0.58 (0.27 to 1.23)	0.15	0.49 (0.21 to 1.11)	0.088
Onset episode severity (VFSS)		54	1.06 (0.86 to 1.31)	0.56	–	–
Time from initial symptoms to corticosteroids	<7 days	41	1.00 (reference)	–	1.00 (reference)	–
	≥7 days	62	1.09 (0.62 to 1.93)	0.76	1.04 (0.59 to 1.85)	0.89
	No treatment	18	1.72 (0.86 to 3.4)	0.12	1.71 (0.81 to 3.62)	0.16
Cumulative IV corticosteroid dose (g)		109	0.96 (0.9 to 1.02)	0.18	–	–
Oral corticosteroid dose (mg/day)		109	0.96 (0.93 to 0.99)	0.0078	0.96 (0.93 to 0.99)	0.014
IVIg or PLEX given as acute immunotherapy	Not given	100	1.00 (reference)	–	–	–
	Given	9	0.8 (0.32 to 1.99)	0.63	–	–

ADEM, acute disseminated encephalomyelitis; EDSS, Expanded Disability Status Score; IVIg, intravenous immunoglobulin; ON, optic neuritis; PLEX, therapeutic plasma exchange; TM, transverse myelitis; VFSS, Visual Functional System Score

### Specifying the optimal dose and duration of oral corticosteroids which delays TTFR

**Figure 2:**
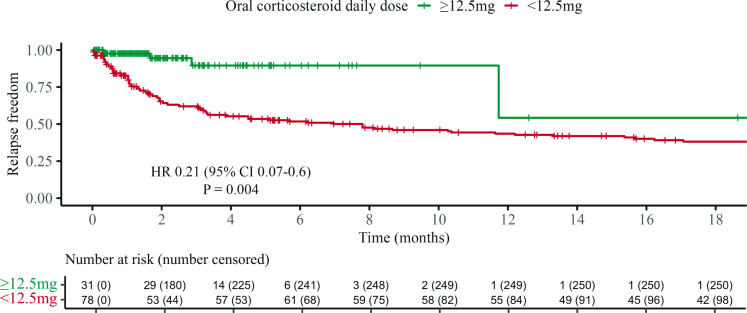
Time to first relapse according to daily oral corticosteroid dose. Simon-Makuch plot of the association of oral corticosteroid dose (as a time-dependent covariate) with time to first relapse, using the dosing threshold of 12.5 mg/day. This plot visualises the comparison of the cumulative hazards of a relapse while patients were treated with oral corticosteroid doses at or above 12.5 mg daily compared with the time they were treated with oral corticosteroid doses lower than 12.5 mg daily. Number censored exceeds total event number because in this analysis, censoring events occur when patients cross the dose threshold and at relapse.

A series of Cox proportional hazard analyses and visualisations were performed to identify the optimal dose and duration for prednisone courses. The association of oral prednisone (as a time-dependent variable) with TTFR was visualised, using a Simon-Makuch plot, which displayed the outcome among patients treated at or above a particular daily oral prednisone dose vs below this dose. We predefined candidate threshold doses ranging from 50 mg/day to 0 mg/day in 2.5 mg/day decrements (representative examples in online supplemental figure 3). The minimum effective dose was identified as 12.5 mg/day in adults (or 0.16 mg/kg/day in children), as this plot demonstrated the best separation of the groups while maintaining precision of the estimate (HR 0.21 (95% CI 0.07 to 0.6); p*=*0.0036; figure 2). Patients treated with oral prednisone dosed ≥12.5 mg/day experienced a 79.1% reduction in the risk of a relapse compared with patients treated with oral prednisone doses <12.5 mg/day, when controlling for age, sex, time to treatment and clinical episode severity.

Next, a similar approach was taken to find the optimal duration to be spent at or above the recommended oral prednisone dose of 12.5 mg/day. A series of Cox proportional hazard analyses and visualisations explored binary cutoffs from 6 months to 0 months in 0.5-month decrements (representative examples in online supplemental figure 4). Based on the separation of the outcomes between the oral prednisone dose groups, the effect estimates and their CIs, the minimum effective duration of oral prednisone dose of ≥12.5 mg/day was identified as 3 months from MOGAD onset (HR 0.15 (95% CI 0.04 to 0.48); p*=*0.0016). This equated to an absolute risk reduction of 42% and a number needed to treat of three to prevent one patient from having relapsed at 12 months.

**Figure 3:**
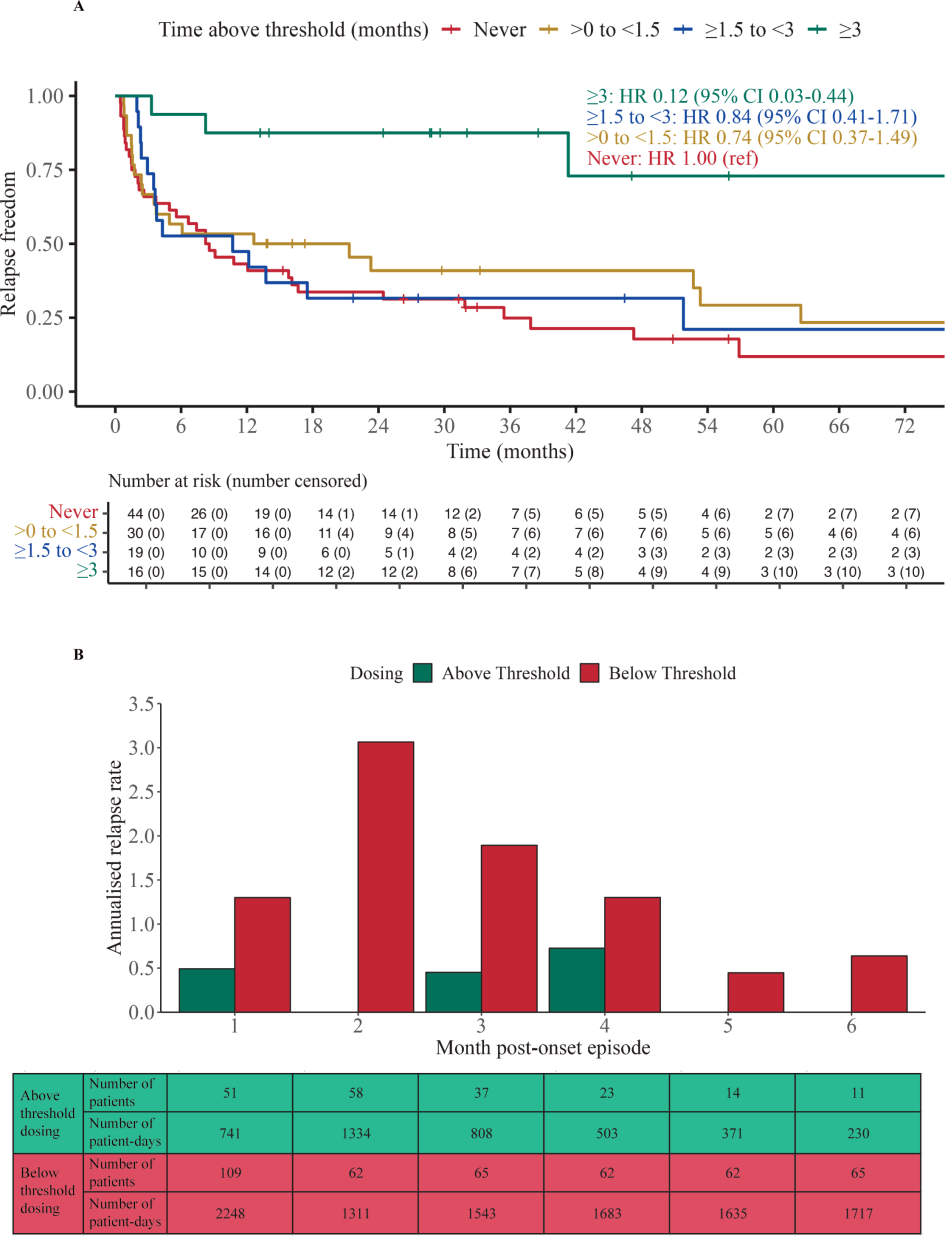
Relapse activity over time since first clinical episode with respect to threshold dosing (≥12.5 mg daily). (**A**) Kaplan-Meier survival time to first relapse curves with patients stratified by time spent above threshold dosing. Adjusted Cox proportional hazard ratios, with ‘never’ stratum as the reference group, are annotated. The median (IQR) cumulative oral corticosteroid dose (mg) for the <1.5 months, 1.5 to <3 months and ≥3 months groups are 1085 (631–1807), 2540 (1722–3538) and 4345 (3761–5965), respectively. (**B**) Annualised relapse rates were compared for each month after onset clinical episode, between patients above threshold dose and those below. Data for each group were aggregated from multiple patient-days in their respective months. The number of patients and patient-days contributing to each group are tabulated.

Effective oral prednisone dosing lasting less than 3 months resulted in a substantially smaller estimated effect size (HR≥0.54) with weaker evidence of an association with TTFR. This point estimate of the ≥3 months persistence on the oral prednisone dose of ≥12.5 mg/day corresponded to an 85.4% reduction in the hazard of a relapse compared with a shorter duration of effective oral prednisone therapy, and an 87.7% reduction (HR 0.12 (95% CI 0.03 to 0.44); p*=*0.0012) when compared with those who never spent any time on effective oral prednisone therapy. Figure 3A visualises this relationship by dividing the cohort into four groups that spent different durations of time at or above the oral prednisone dose of 12.5 mg/day. The median (IQR) cumulative oral prednisone dose (mg) for the <1.5 months, 1.5 to <3 months and ≥3 months groups were 1085 (631–1807), 2540 (1722–3538) and 4345 (3761–5965), respectively. The superiority of the ≥3 months group was maintained in both paediatric and adult subgroups (online supplemental figure 5). Analysis of patients diagnosed in epochs before 2014, between 2014 and 2018 and after 2018 did not reveal any significant differences in either oral corticosteroid dose or duration.

To evaluate the optimal duration of oral prednisone treatment using a different approach, annualised relapse rates were compared during the time when patients were treated with ≥12.5 mg/day oral prednisone and <12.5 mg/day oral prednisone during each of the initial 6 months after onset (figure 3B). The differences in annualised relapse rate in patients in these two groups were largest in the first 3 months (0.81, 3.06 and 1.44 relapses, respectively) and consistently smaller thereafter (0.58, 0.45 and 0.64). This suggests 3 months might be an adequate duration for this regimen, without marked additional benefits with longer durations of corticosteroids.

**Figure 4:**
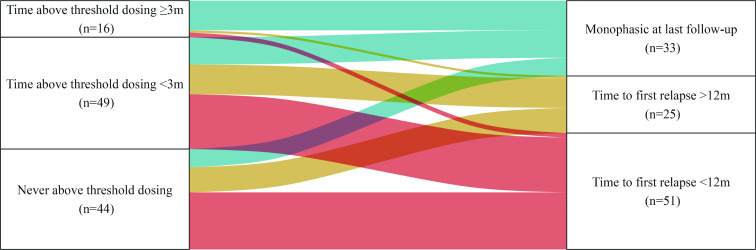
Disease course outcome according to time above threshold dosing. Alluvial plot of the disease course outcome for patients with different durations spent above threshold dosing of oral corticosteroids (≥12.5 mg/day) for their initial episode of MOGAD. 13/17 (76%) of patients remaining above threshold dosing for more than 3 months had not experienced a relapse at last follow-up, compared with 12/48 (25%) of those who spent time above threshold dosing that was less than 3 months, and 8/45 (18%) of those who never received above threshold dosing. MOGAD, myelin oligodendrocyte glycoprotein antibody-associated disease.

Finally, the influence of oral corticosteroid treatment at disease onset and the likelihood of exhibiting a monophasic disease course was evaluated. MOGAD remained monophasic in 13/16 (81.3%) patients who spent ≥3 months treated with ≥12.5 mg/day oral corticosteroids, compared with 12/49 (24.5%) of patients who were treated with ≥12.5 mg/day oral corticosteroids for less than 3 months, and 8/44 (18.2%) of patients who were never treated with ≥12.5 mg/day oral corticosteroids (figure 4, p<0.001).

### Adverse events from corticosteroid exposure during onset clinical episode

Adverse events related to prednisone exposure during the onset episode were reported in 8/65 (12.3%) patients. These included six grade 1 events, one grade 2 event and one grade 3 event (table 3). There were no grade 4 or 5 events reported. The one grade 3 event occurred in a patient who had been on 8 months of oral prednisone (cumulative dose 5574 mg), and who experienced cholecystitis requiring a cholecystectomy. We note that long-term steroid-related adverse events associated with the treatment of subsequent relapses with oral prednisone were documented in some patients (online supplemental table 1).

**Table 3:** Adverse events in patients exposed to oral corticosteroids (n*=*65)

CTCAE	Number (% of patients exposed to oral corticosteroids)	Type	Median cumulative oral corticosteroid dose (mg)
1	6 (9.2)	5 Cushingoid appearance; 2 Irritability; 1 hyperglycaemia; 1 HTN worsening	5583 (IQR 4158–6395)
2	1 (1.5)	Weight gain, severe acne and insomnia requiring medication	1750
3	1 (1.5)	Cholecystitis requiring surgery	5574
4	0	–	–
5	0	–	–

Data are summarised as n (%), or median (IQR).

CTCAE, Common Terminology Criteria for Adverse EventsHTNhypertension

## Discussion

Factors at MOGAD onset which influence the induction and maintenance of disease remission remain to be fully elucidated. MOGAD patients at disease onset may be prone to ‘very early relapses’ (<3 months after onset) and ‘delayed early relapses’ (3–12 months after onset).^14^ A recent publication found an association of very early relapses in adults, and delayed early relapses in children and adults, with an increased risk of long-term relapsing disease.^14^ This provides a compelling argument for effective early treatment after onset to modify long-term disease course.

A key historic study related to the duration of corticosteroid treatment following ON in 448 adults, the Optic Neuritis Treatment Trial, identified a 14-day course of oral prednisone was associated with a higher risk of further ON compared with a group receiving 3 days of intravenous methylprednisolone and 11 days of oral prednisone, or placebo.^28^ However, subsequent evaluation of 177 patients’ available sera from this trial identified only three were seropositive for MOG antibodies.^29^ A publication from 2019 surveying 52 neurologists from 22 countries revealed that there was significant equipoise with respect to clinical practice at disease onset in MOGAD.^16^ All clinicians surveyed used pulsed intravenous methylprednisolone for acute clinical episodes of MOGAD. Of interest, our univariate analyses showed that the use of intravenous corticosteroids did not appear to influence the risk of a relapse. 60% usually or always prescribed ≥3 months course of oral corticosteroid therapy, 23% sometimes did and 17% rarely or never did.^16^ Paediatric neurologists were more likely to never have treated with ≥3 months of corticosteroids compared with adult neurologists. The duration of preferred oral corticosteroid treatment varied significantly and ranged from less than 3 months to ≥18 months.^16^

Our cohort is of value as it included patients with clinical onset before widespread knowledge of MOGAD as a distinct syndrome, with 18 patients who did not receive any immunotherapy for their first clinical episode, enabling direct comparisons with a ‘never treated’ at onset group. Additionally, the current study reiterates our prior findings that up to 55% of relapsers experiencing their first relapse, do so on prednisone doses <10 mg/day or within 10 weeks of cessation of prednisone.^3 4^

MOGAD episodes have been demonstrated to be extremely responsive to corticosteroids with prior studies demonstrating varying durations of corticosteroid treatment ranging from 2 weeks to 6 months identified as being associated with a reduced propensity to relapses.^34 9 11^,^13^ There are publications which query the role of corticosteroids in children beyond a short intravenous course at disease onset,^30 31^ while others identified that corticosteroid treatment for ≥5 weeks in children was associated with a lower risk of relapse.^12^ A prospective cohort study of 276 patients revealed an HR of 0.33 for developing a relapsing course in those treated with prednisone as a maintenance therapy, compared with those who were not.^32^ However, there remains a lack of consensus on the optimal dose and duration of corticosteroids at MOGAD onset.

There is accumulating evidence of increased relapse-associated disability in MOGAD, particularly with visual and sphincter dysfunction, reinforcing relapse prevention remains a critical therapeutic goal.^4 9 12 33^ While relapse rates are reported as 30%–60% in MOGAD,^8 11 13 34 35^ this may be in the order of 70% when patients are followed up for over 5 years,^9^ as in our cohort. This brings to question whether there is a role for introducing corticosteroid-sparing maintenance immunosuppression at disease onset in order to minimise relapse-associated deficits as well as corticosteroid exposure. However, while treatment of MS and NMOSD highlights the need for ongoing immunotherapy from diagnosis, in MOGAD there remains a proportion of patients who have a monophasic illness who may be unnecessarily exposed to long-term immunosuppression if this approach was uniformly adopted at disease onset.

The most salient finding of this study is the 79% reduction in the hazard of relapse at or above a dose threshold of prednisone 12.5 mg/day (0.16 mg/kg/day for children), compared to those below this dose threshold. The other key finding was strong evidence of an 88% reduced hazard of relapse for those spending at least 3 months at or above this threshold compared to those that never received this dosing. Furthermore, the annualised relapse rate reduction was not as striking when the prednisone regimens extended beyond 3 months. While many patients are commenced initially on intravenous methylprednisolone or high doses of prednisone in the range of 1 mg/kg/day, our findings suggest that this high dose may be rapidly reduced to our recommended dose of 12.5 mg/day in adults (0.16 mg/kg/day in children) for 3 months, in order to minimise total oral corticosteroid exposure while still maintaining relapse freedom. Given there were 16 patients in the ≥3 months treatment group, further prospective studies, with larger cohorts following the proposed dosing and duration protocol, will be essential to corroborate our findings.

None of the patients in this cohort on prednisone were documented to have CTCAE grade 4 or 5 adverse events related to their onset treatment. No patient experienced a CTCAE grade >2 adverse event with a cumulative dose of <5000 mg of oral prednisone. The suggested optimal dose and duration at onset that our results recommend accumulates to a total dose that resides below this limit. Moreover, the reduction in future relapses evident in our study also would be expected to have a corticosteroid-sparing effect, by avoiding high cumulative doses associated with treatment of relapses.^15^

Our study is retrospective, with inherent limitations such as the potential for selection bias, treatment indication bias, informative censoring and unmeasured confounding factors. Our statistical models were refined to adjust for known confounders. Neurological and visual assessments were not standardised across centres due to the retrospective nature of this study. It is likely that among patients with a documented monophasic disease course, some will develop a relapsing course given sufficient time. This, however, does not affect our primary finding of TTFR, or treatment recommendations within the first 12 months after onset. In order to analyse paediatric and adult patients concurrently, we weight-adjusted doses of oral prednisone. While this is considered a reasonable conversion method,^21^ and weight-based dosing is an established convention in paediatric medicine, we acknowledge that adult body weight data for all patients in this retrospective study were not available for inclusion in this analysis. Addressing immortal time bias presented an analytical challenge, especially when assessing a time-based outcome (TTFR), with the exposure stratified by a time based criterion (time above threshold dosing). In our approach, we accounted for all above-threshold dosing durations, even when this extended past the point of the first relapse, to reflect the intention to treat with a certain oral prednisone course. Finally, documentation of adverse events following prednisone exposure, while likely accurate for severe short-term adverse events, could underestimate more minor adverse events and the long-term consequences of corticosteroid use for the onset episode.

This study provides evidence supporting the use of oral corticosteroids at the onset of MOGAD. We propose evidence-based recommendations highlighting that patients at disease onset should be treated with a minimum effective dose of 12.5 mg daily of oral prednisone (0.16 mg/kg/day for children) for a minimum duration of 3 months in order to delay TTFR and minimise cumulative corticosteroid exposure.

## supplementary material

10.1136/jnnp-2024-333463 online supplemental file 1

## Data Availability

Data are available on reasonable request.
