# Supplementary material for: Oral corticosteroid dosage and taper duration at onset in myelin oligodendrocyte glycoprotein antibody-associated disease influences time to first relapse
Source: J Neurol Neurosurg Psychiatry. 2024 May 14;95(11):e333463. doi: 10.1136/jnnp-2024-333463 (PMC11503134; doi:10.1136/jnnp-2024-333463)
Supplement: online supplemental file 1 [file jnnp-95-11-s001.pdf]

Supplemental Online Content

Supplementary Figure 1. Relationship between oral corticosteroid treatment and relapses
Supplementary Figure 2. Temporal dynamics of prednisone dosing for all treated patients
Supplementary Figure 3. Representative examples of a series of Simon-Makuch plots in descending thresholds to find the optimal oral corticosteroid dose
Supplementary Figure 4. Representative examples of a series of Kaplan-Meier plots in descending duration cutoffs above threshold dose to find the optimal duration of above threshold dosing
Supplementary Figure 5. Time to first relapse stratified by months spent above threshold oral corticosteroid dose in paediatric (A) and adult patients (B)
Supplementary Table 1. Long term adverse events possibly related to corticosteroids
Supplementary References

Supplementary Figure 1. Relationship between oral corticosteroid treatment and relapses

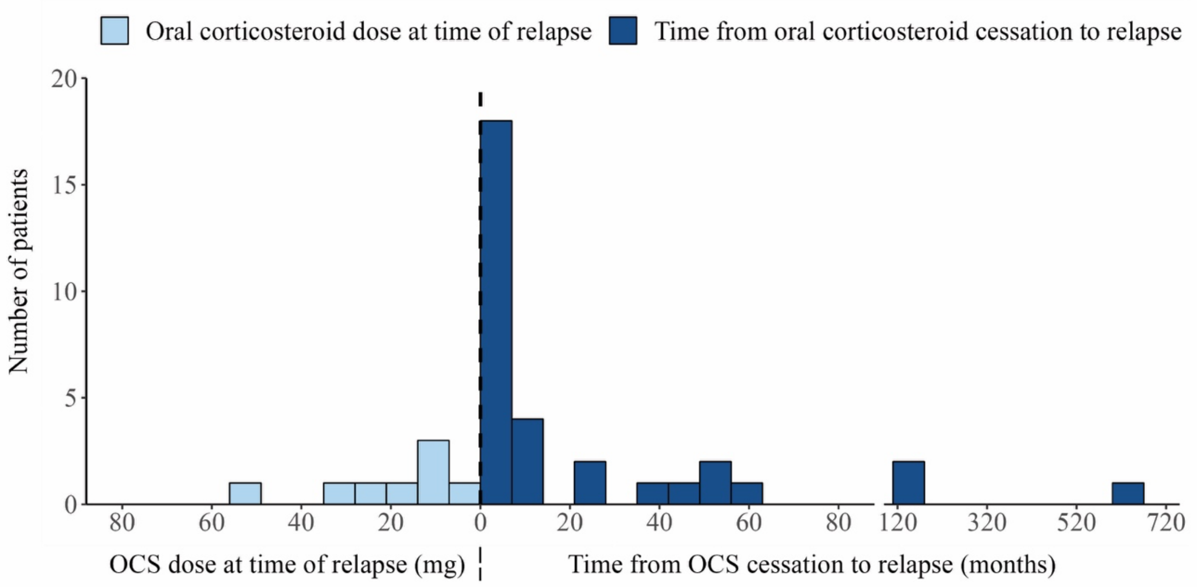

Histogram of the relationship between oral corticosteroid course and relapse in patients who relapsed and had been exposed to oral corticosteroids (n=40). The x-axis left of the dashed vertical line indicate the oral corticosteroid dose at which patients relapsed, and the values to the right indicate the time from oral corticosteroid cessation to relapse. There is a break in the x-axis from 80-120 months with a 10-fold change in scale above 120 months to include three outliers with very late relapses. OCS, oral corticosteroid.

Supplementary Figure 2. Temporal dynamics of prednisone dosing for all treated patients

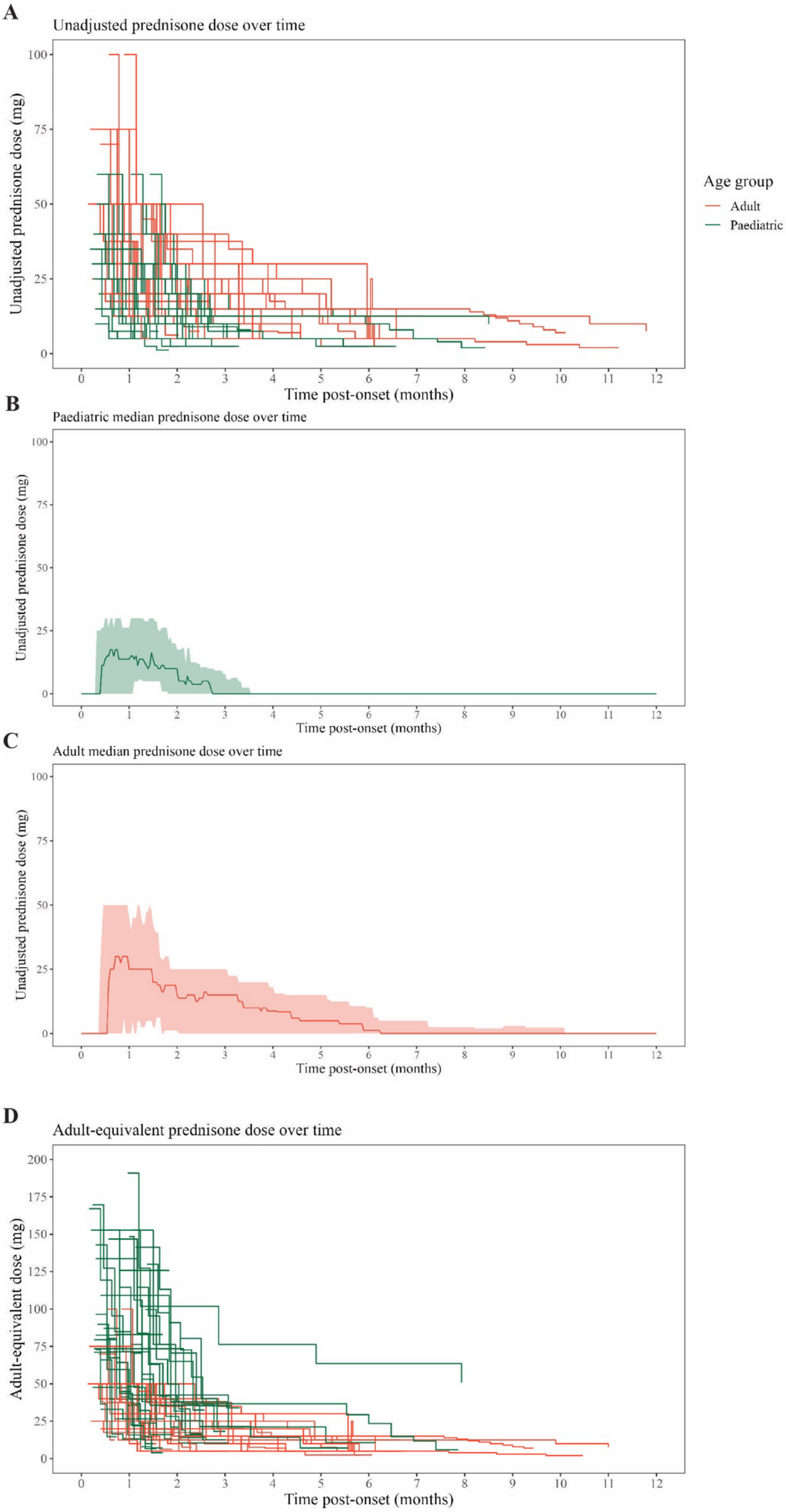

Prednisone dose over the first year for each of the patients treated with oral corticosteroids (n=65, 60%) for their first clinical episode of MOGAD is indicated above. Raw doses are provided (A) stratified by age. The median (solid line) and interquartile range (shading) dose for prednisone over time is shown for paediatric patients (B) and adult patients (C). Panel A is then re-presented with the paediatric doses weight-adjusted to adult equivalent doses (D), as used in our analysis. The treated patients in our cohort were given corticosteroids for a median (IQR) of 47 days (30-140), with an initial adult equivalent dose of 73 mg (50-100). There was no statistically significant difference in the mean total prednisone dose between females and males (p-value 0.81).

Supplementary Figure 3. Representative examples of a series of Simon-Makuch plots in descending thresholds to find the optimal oral corticosteroid dose

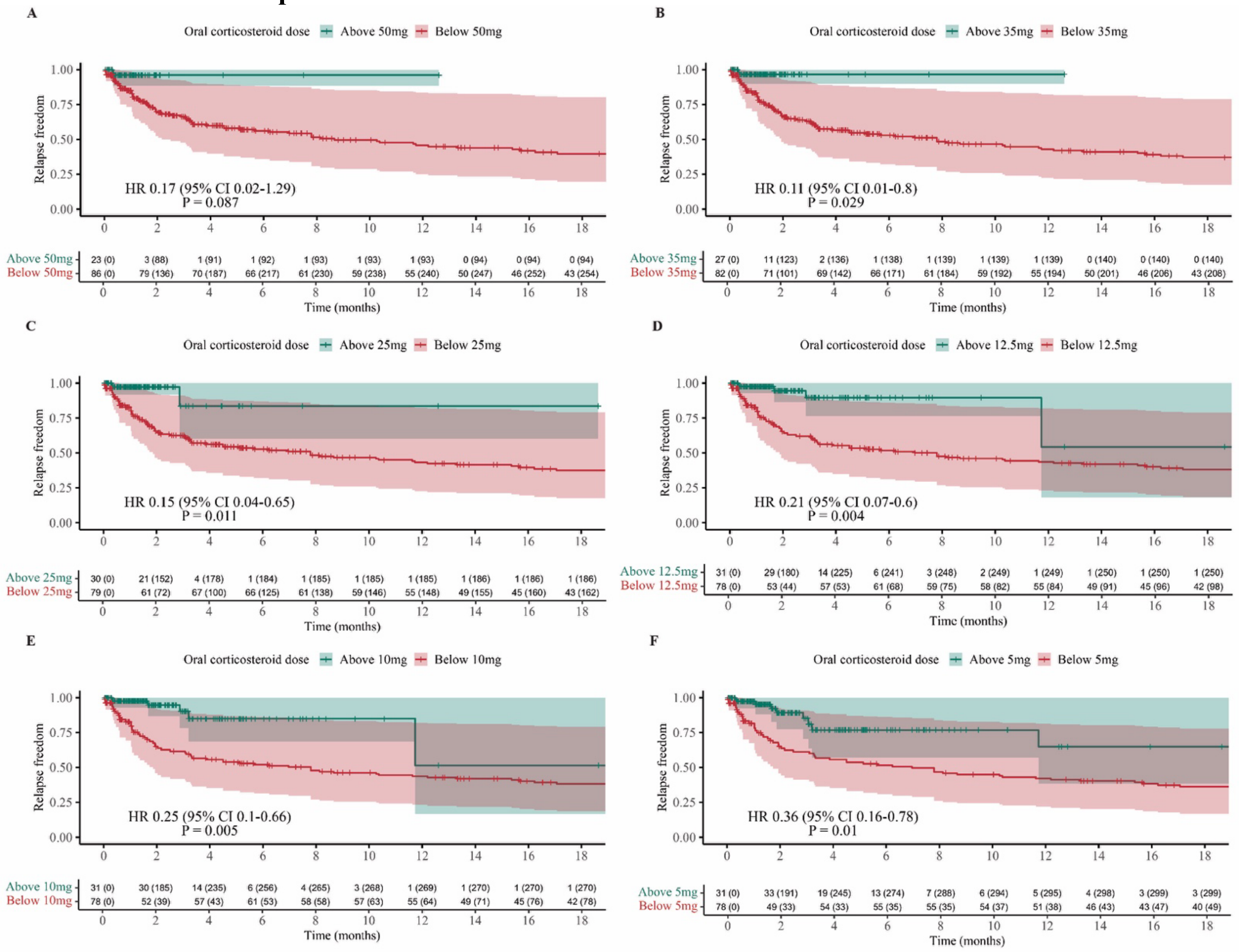

These six survival plots are representative of a series performed with the dosing cutoff reducing by 2.5 mg/day from 50 mg/day to 0 mg/day. They are presented in descending order from 50mg/day dosing cutoff in the top left of the figure (A) down to 5mg/day in the bottom right (F). The shading represents the 95% confidence interval. In Simon-Makuch plots of time-varying covariates, the number of subjects at risk is very small in at least one stratum at early time points, therefore it is common practice to move the starting time point from study time 0 to the median time to switch membership of groups (Day 13)[1]. The number of censored observations in these plots is larger than the total number of patients in the study as censoring events occur at last follow up for those who did not experience the event (a relapse) and when patients cross the dosing threshold. The survival curve and the annotation are confounder-adjusted effect estimates. We identified 12.5mg/day as the optimal dosing threshold as the separation between the two lines over the follow-up period, and the confidence interval are the most favourable at this dose cutoff. The x-axis range is 18 months as there is insufficient data for patients on any continuous dose of oral corticosteroids beyond this point. CI, confidence interval; HR, hazard ratio.

Supplementary Figure 4. Representative examples of a series of Kaplan-Meier plots in descending duration cutoffs above threshold dose to find the optimal duration of above threshold dosing

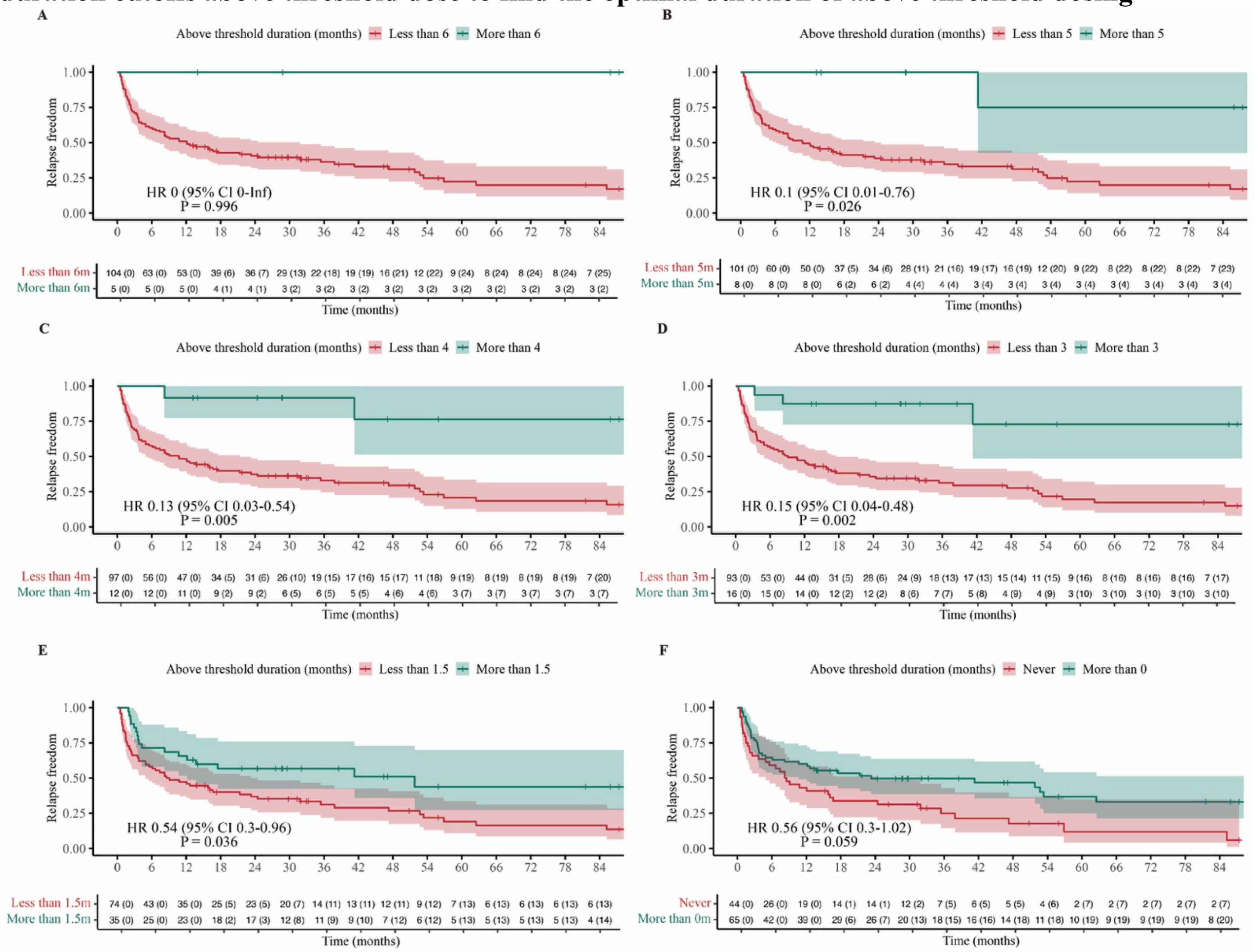

These six survival plots are representative of a series performed with the dosing duration cutoff reducing by 0.5 month from 6 months to 0 months. They are presented in descending order from 6 months duration cutoff in the top left of the figure (A) down to 0 months in the bottom right (F). The shading represents the 95% confidence interval. The annotation is the confounder-adjusted effect estimate. We identified three months as the optimal threshold dosing duration as the separation between the two groups over the follow-up period, and the confidence interval are the most favourable at this duration cutoff. CI, confidence interval; HR, hazard ratio.

Supplementary Figure 5. Time to first relapse stratified by months (m) spent above threshold oral corticosteroid dose in paediatric (A) and adult patients (B)

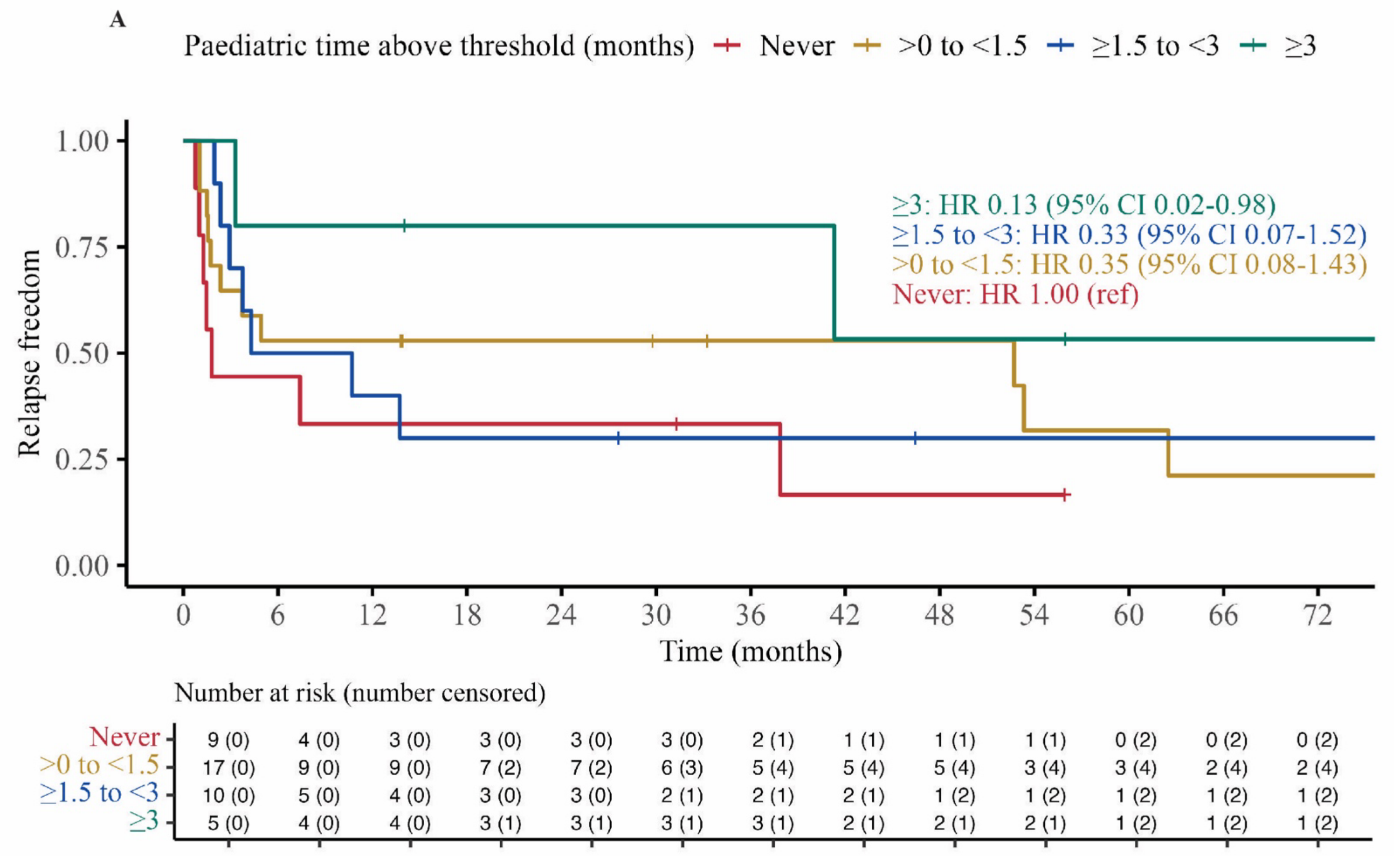

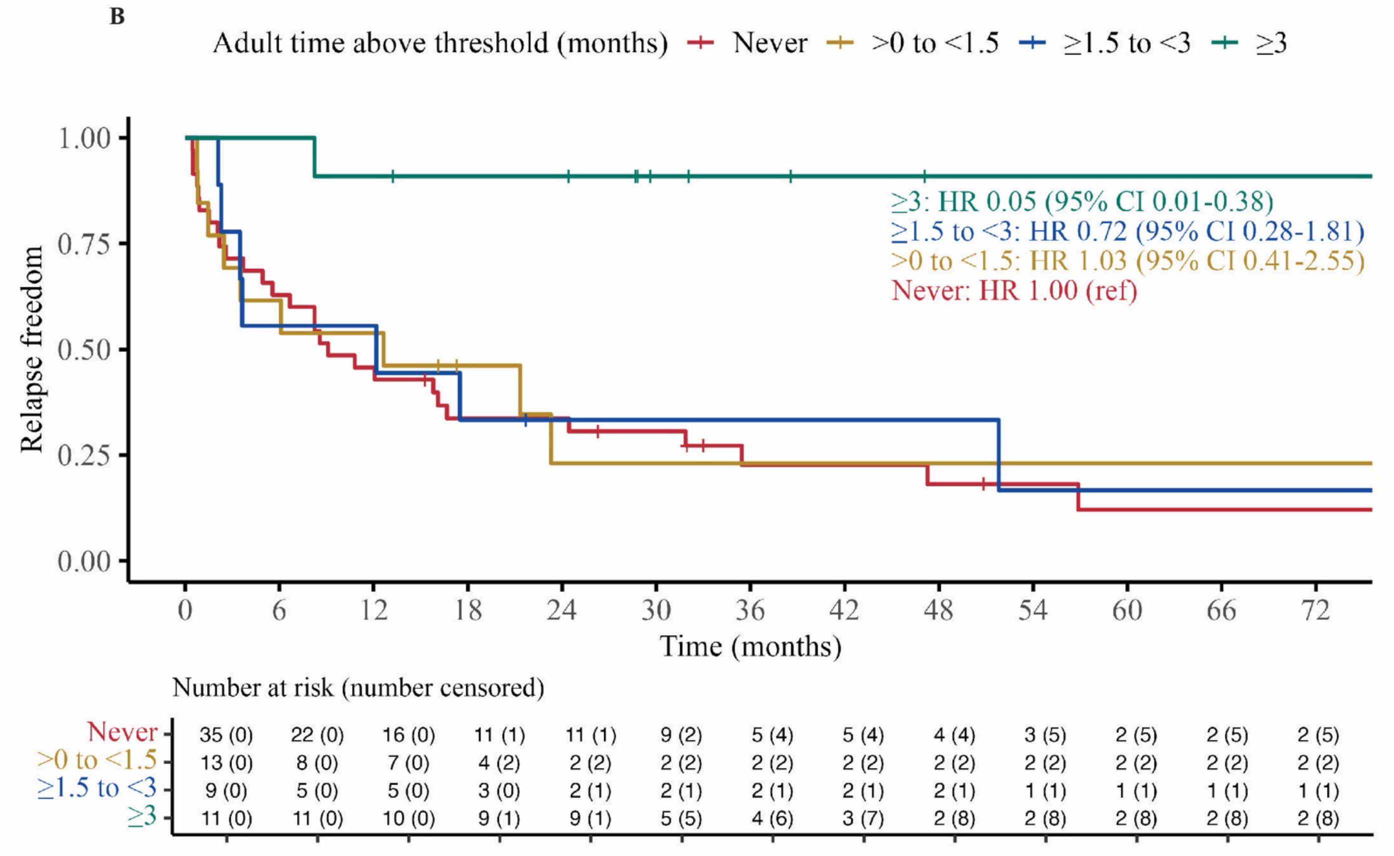

Kaplan-Meier survival curves for paediatric patients (A) and adult patients (B) of time to first relapse according to time spent above threshold dosing (≥12.5mg/day). Adjusted Cox proportional hazard ratios are annotated next to their respective curves. The "Never" stratum who never received oral corticosteroids above threshold dosing, are the reference group. CI, confidence interval; HR, hazard ratio.

Supplementary Table 1. Long term adverse events possibly related to corticosteroids

Timing relative to onset episode	Adverse event severity (CTCAE)	No. of patients	Adverse event (AE) descriptions (Possibly related to corticosteroids)	Cumulative IV corticosteroid dose [Median (Q1-Q3)] for the clinical episode associated with the AE (grams)	Cumulative prednisone dose [median (Q1-Q3)] for the clinical episode associated with the AE (grams)	Days above threshold dose [median (Q1-Q3)] of prednisone for the clinical episode associated with the AE
Onset episode	No adverse events	98	-	5.77 (3.75-7.19)	1.83 (1.05-3.67)	18 (0-47)
Onset episode	1	7	Irritability; Steroid-induced hyperglycaemia; Cushingoid appearance; Weight gain and mild irritability; Slightly cushingoid and mild worsening of HTN; Mild cushingoid appearance; Weight gain and irritability	9.54 (8.66-12.66)	5.58 (4.16-6.39)	78 (47-152)
Onset episode	2	3	Anxiety and paranoia; Weight gain, Severe acne and insomnia; Primary VZV infection	3.75 (3.75-5.81)	1.75 (1.75-1.75)	0 (0-22)
Onset episode	3	1	Cholecystitis requiring cholecystectomy	6.25 (6.25-6.25)	5.57 (5.57-5.57)	154 (154-154)
Onset episode	4	0	-	-	-	-
Onset episode	5	0	-	-	-	-
Relapses <3y	No adverse events	59	-	5.96 (3.75-7.82)	5.34 (2.53-8.48)	14 (0-138)
Relapses <3y	1	7	Irritability; Insomnia, irritability; Irritability, acne; "Some undesirable effects"; Significant weight gain; Osteopaenia; Irritability with IV steroids	6.25 (5.62-6.25)	3.19 (1.85-4.52)	69 (12-99)
Relapses <3y	2	4	Osteopaenia; Osteopenia and central adiposity; Irritability and insomnia; Obstructive sleep apnoea	3.75 (3.38-3.75)	17.48 (11.34-22.63)	237 (61-534)
Relapses <3y	3	1	Bilateral cataracts requiring surgery	3.75 (3.75-3.75)	8.81 (8.81-8.81)	343 (343-343)
Relapses <3y	4	0	-	-	-	-
Relapses <3y	5	0	-	-	-	-

Relapses ≥3 to <5y	No adverse events	17	-	6.25 (3.93-8.58)	1.73 (0.34-3.58)	2 (0-27)
Relapses ≥3 to <5y	1	4	Weight gain and irritability; Dyspepsia, irritability, insomnia; Irritability; Weight gain; Weight gain	8.59 (6.17-9.07)	2.47 (1.33-12.56)	55 (34-366)
Relapses ≥3 to <5y	2	1	Osteopaenia	6.09 (6.09-6.09)	4.10 (4.10-4.10)	0 (0-0)
Relapses ≥3 to <5y	3	0	-	-	-	-
Relapses ≥3 to <5y	4	0	-	-	-	-
Relapses ≥3 to <5y	5	0	-	-	-	-
Relapses ≥5y	No adverse events	24		3.75 (3.75-6.25)	1.19 (0.59-3.83)	0 (0-18)
Relapses ≥5y	1	5	Anxiety, nausea and insomnia; Dyspepsia; Weight gain, irritability; Dyspepsia, mood changes and insomnia; Dyspepsia, irritability and insomnia with IV steroids; Acne; Mild central adiposity	3.75 (2.50-4.38)	2.20 (1.91-5.17)	39 (2-62)
Relapses ≥5y	2	8	Osteopaenia; Cholelithiasis requiring surgery; Osteopaenia; Obesity; Weight gain and headache; Osteopaenia; Glaucoma; Weight gain; HTN	3.75 (3.75-3.75)	7.44 (5.02-14.87)	21 (0-161)
Relapses ≥5y	3	0	-	-	-	-
Relapses ≥5y	4	1	Depression and suicidality	6.25 (6.25-6.25)	3.35 (3.35-3.35)	35 (35-35)
Relapses ≥5y	5	0	-	-	-	-

CTCAE = Common terminology for criteria for adverse events; HTN = hypertension; Q = quartile; VZV = varicella zoster virus; y = years.

Supplementary References

1. Schultz LR, Peterson EL, Breslau N. Graphing survival curve estimates for time-dependent covariates. *Int J Methods Psychiatr Res*. 2002;11(2):68-74. doi:10.1002/mpr.124
